# The *KIR2DL1* intermediate upstream element participates in gene activation

**DOI:** 10.1007/s00251-023-01321-9

**Published:** 2023-10-06

**Authors:** Paul W. Wright, Hongchuan Li, Md Ahasanur Rahman, Erik M. Anderson, Megan Karwan, Jeffrey Carrell, Stephen K. Anderson

**Affiliations:** 1 Basic Science Program, Frederick National Laboratory for Cancer Research, Frederick, MD 21702 USA; 2 Cancer Innovation Laboratory, Center for Cancer Research, NCI, Frederick, MD 21702 USA

**Keywords:** NK cells, KIR2DL1, Transcription, CRISPR, YTS

## Abstract

The human *KIR* genes encode a family of class I MHC receptors that are expressed on subsets of NK cells. The expression of KIR proteins is controlled by a stochastic process, and competition between sense and antisense promoter elements has been suggested to program the variegated expression of these genes. Previous studies have demonstrated distinct roles of distal, intermediate, and proximal sense promoter/enhancer elements in gene activation and expression. Conversely, proximal and intronic antisense promoter transcripts have been associated with gene silencing at different stages of NK cell development. In the current study, we examine the effect of intermediate promoter deletion on KIR2DL1 expression in the YTS cell line. Homozygous deletion of the *KIR2DL1* intermediate element did not affect proximal promoter activity but resulted in increased detection of upstream transcripts. No significant changes in alternative mRNA splicing or expression levels of KIR2DL1 protein were observed. However, intermediate element deletion was associated with a reduced frequency of gene activation by 5-azacytidine. Taken together, these results indicate that the intermediate element is not an enhancer required for KIR expression; however, it is required for the efficient activation of the gene.

## Introduction

Natural killer (NK) cells are lymphoid cells of the innate immune system that can eradicate stressed cells, such as virus-infected and tumor cells, without prior sensitization (Hamerman et al. 2005). The cytolytic activity of NK cells is controlled by receptors that interrogate potential target cells for either aberrant or missing major histocompatibility complex (MHC) class I molecules on their surfaces (Yokoyama 1998; Parham and Guethlein 2018). The predominant category of MHC class I receptors expressed by human NK cells is derived from the killer cell immunoglobulin-like receptor (KIR) family of genes (Yawata et al. 2002). Fourteen *KIR* genes and two pseudogenes have been identified and mapped to human chromosome 19q13.4 (Trowsdale et al. 2001). Furthermore, the *KIR* gene cluster has been shown to exhibit a remarkable diversity with respect to gene content and alleles (Middleton and Gonzelez 2010). The human KIR proteins are a family of type I transmembrane glycoproteins that are expressed probabilistically by mature NK cells and a small percentage of T cells (Moretta et al. 1990; Valiante et al. 1997; Yawata et al. 2008). The KIR family of MHC class I receptors possesses a repertoire of both inhibitory and activating receptors which distinguish between normal host MHC class I expression versus missing or modified class I molecules that arise during neoplasia and viral pathogenesis. The net balance of reciprocity between these inhibitory and activating signals ultimately determines the fate of the target cell (Purdy and Campbell 2009).

**Fig. 1:**
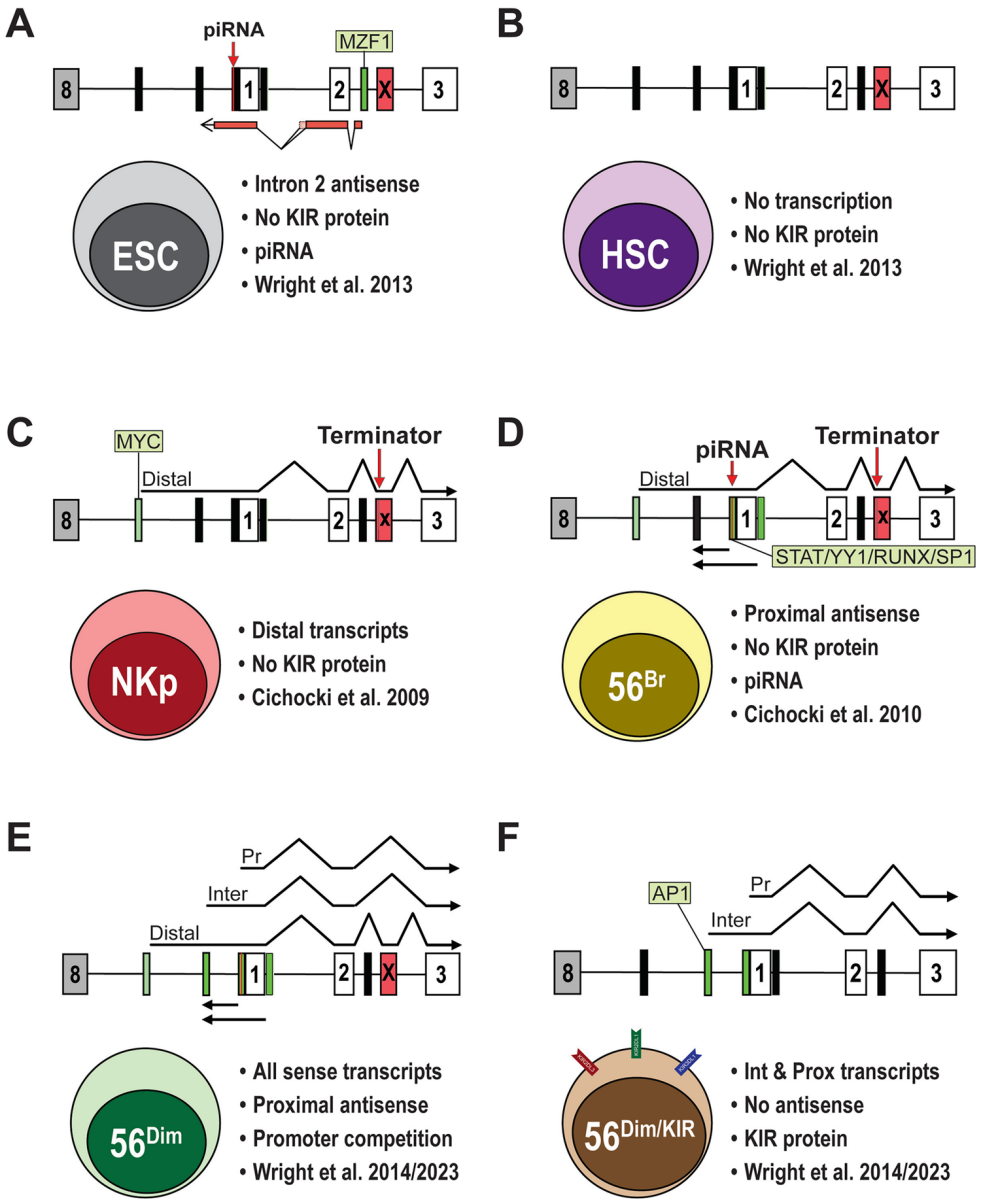
Promoter activity in the 5′ regulatory region of the *KIR* genes during NK cell development. **A** Schematic illustrating intron 2 antisense transcription in embryonic stem cells (ESC). The complete *KIR* regulatory region from the polyA site of the final exon of the preceding gene (gray rectangle–8) to exon 3 (white rectangle–3) is shown. Black filled rectangles indicate silent promoters, and the green rectangle represents the active intron 2 antisense promoter. The light green box indicates the importance of MZF1 for promoter activity. The antisense transcripts are shown below, with lines indicating splicing events. The location of the piRNA generated from the antisense strand is indicated by the red arrow. An alternative exon containing an in-frame stop codon is indicated by the red rectangle labeled with an x. **B** Schematic illustrating the lack of transcription in CD34 hematopoietic progenitors (HSC). **C** Schematic illustrating distal transcription in NK progenitors (NKp). The active distal promoter is indicated by a green rectangle, and an untranslatable alternatively spliced mRNA containing exon 2a (x) is shown above. The position of a termination codon is indicated by the red arrow. The light green box indicates the importance of MYC for distal promoter activity. **D** Schematic illustrating the presence of distal sense and proximal antisense in immature CD56^bright^ NK cells (CD56^Br^). Unspliced antisense transcripts are indicated below. The light green box indicates the importance of STAT-, YY1-, RUNX-, and SP1-binding sites for proximal promoter activity. **E** Schematic illustrating the presence of multiple sense and antisense transcripts in KIR-ve CD56^dim^ NK cells. Distal, intermediate (Inter), and proximal (Pr) sense transcripts are indicated above. **F** Schematic illustrating the presence of intermediate and proximal sense transcripts in mature CD56^dim^ KIR^+ve^ mature NK cells (CD56^dim/KIR^). The light green box indicates the importance of AP1 for intermediate promoter activity

*KIR* gene expression has been shown to be variegated, resulting in NK cells with various combinations of specific *KIR* genes, with most expressing between 1–3 KIR (Yawata et al. 2008; Li et al. 2008). The mechanisms which regulate the probabilistic expression of *KIR* genes are not fully understood. However, evidence suggests that interaction of sense RNA transcripts originating from distal promoters with antisense transcripts from the bidirectional *KIR* proximal promoter or an intronic antisense promoter may play a significant role (Fig. 1; Anderson 2014; Pascal et al. 2006; Stulberg et al. 2007; Davies et al. 2007). The *KIR* loci are initially silenced in stem cells by a noncoding antisense transcript originating within intron 2 that splices into the first exon, producing an antisense transcript covering the promoter region and ending at a polyA site ~ 300 bp upstream of exon 1. MZF1 was identified as a key transcription factor driving the stem cell-specific activity of the intron 2 antisense promoter (Fig. 1A; Wright et al. 2013). Thus, no KIR transcription can be detected in CD34 hematopoietic progenitors (Fig. 1B). Non-translatable distal *KIR* transcripts first appear in NK precursors potentially activating the downstream bidirectional proximal *KIR* promoter and subsequentially competing/interacting with antisense transcripts to determine whether the gene is silenced or remains in a state capable of protein expression. The *KIR* distal promoter contains a MYC-binding site, and overexpression of MYC in developing NK cells has been shown to augment both transcription from this promoter and the frequency of NK cells that express KIR (Fig. 1C, D; Cichocki et al. 2009). Antisense transcripts from the proximal *KIR* promoter are ~ 300 bp, unspliced, and polyadenylated and can be detected in developing NK cells up to the CD56^+^ dim/KIR^−^ stage. The antisense lncRNAs have been shown to silence the *KIR* proximal promoter through the formation of a 28 bp Piwi RNA (piRNA) which mediates gene silencing (Fig. 1A, D, E; Cichocki et al. 2010). An additional intermediate promoter region located ~ 250 bp upstream of the proximal promoter was identified and shown to produce translatable mRNA transcripts (Fig. 1E, F; Wright et al. 2014). Once KIR protein expression is initiated, antisense transcripts are no longer detectable in CD56^+^ KIR^+^ cells (Fig. 1F).

*KIR2DL4* is a unique member of this gene family, as it is not expressed in a variegated manner, and all mature CD56^+^ NK cells express KIR2DL4 (Rajagopalan and Long 1999). The *KIR2DL4* gene contains two distal promoters 900 bp and 11 kb upstream of the bidirectional proximal promoter (Stulberg et al. 2007). Transcripts originating from both distal promoters are spliced to 190 bp upstream of the start codon. The distal location of *KIR2DL4* promoters prevents the formation of promoter-associated dsRNA from the proximal reverse transcripts and subsequent gene silencing (Stulberg et al. 2007). Therefore, studies conducted thus far suggest that sense transcripts originating from both distal and proximal *KIR* promoters are essential for gene expression while the presence of antisense transcription is indicative of gene silencing.

A study of individuals with low KIR2DL1 expression led to the identification of a novel *KIR2DL1* allele (KIR2DL1*0040115) with a cluster of three single nucleotide polymorphisms (SNPs) in the distal promoter region (Wright et al. 2014). Analysis of the three SNPs revealed that they create a ZEB1-binding site near the MYC/USF-binding site that was associated with a significant reduction in distal transcription. Paradoxically, proximal *KIR2DL1* transcripts from these individuals increased, but surface expression of KIR2DL1 was diminished, and a direct correlation of expression with levels of distal transcript was observed. Transcripts from the KIR2DL1*0040115 allele showed increased inclusion of alternative exons with stop codons. The *KIR2DL1* distal element may act as an enhancer, as suggested by our earlier findings which demonstrated that individuals with *KIR2DL1* alleles containing ZEB1 SNPs exhibited decreased transcription from both distal and intermediate promoters. To address the hypothesis that the translatable intermediate *KIR2DL1* transcripts are the primary driver of protein expression, a 281 bp region containing the intermediate promoter region was deleted in YTS cells using CRISPR. In contrast to the ZEB1 mutation, deletion of the intermediate promoter region did not decrease distal transcript levels; however, it did result in decreased efficiency of *KIR2DL1* gene activation without decreased proximal transcription.

## Methods

### NK cell isolation

Healthy volunteers were recruited through the NCI-Frederick Research Donor Program (http://ncifrederick.cancer.gov/programs/science/rdp/default.aspx). The *KIR* genotype of each donor was determined as previously described (Martin and Carrington 2008). Donors possessing only the KIR2DL1*003 allele were chosen to match the allele present in the YTS cell line. NK cells were separated from the peripheral blood of healthy donors by Histopaque (Sigma-Aldrich) gradient centrifugation using the RosetteSep Human NK Cell Enrichment Cocktail (STEMCELL Technologies).

### Cell culture

YTS cells were cultured in RPMI 1640 medium supplemented with 10% FBS (Hyclone), 100 units/ml penicillin, 100 µg/ml streptomycin, and 2 mM L-glutamine (Gibco) at 37 °C and 5% CO_2_. Cells were passaged every 3–4 days and plated at 3 × 10^5^ cells/ml in 75 cm^2^ flasks. The KIR^+^ cell line was derived by treating parental YTS with 5-azacytidine transiently for 72 h followed by washing and staining with anti-KIR2DL1 antibody. The KIR2DL1-expressing cells were purified by three rounds of FACS sorting until a stable line was generated.

### CRISPR/Cas 9 deletion of the KIR2DL1 intermediate promoter

The following oligonucleotide sequences were used as CRISPR guides to delete a 281 bp region containing the *KIR2DL1* intermediate promoter/enhancer region:

3′ Guide-For5′-**CACCG**TTAGGCATCTCGTGTTCGGG-3′

3′ Guide-Rev5′-**AAAC**CCCGAACACGAGATGCCTAA**C**-3′

5′ Guide-For5′-**CACCG**AAACTCCAGAATTTACAGGT-3′

5′ Guide-Rev5′-**AAAC**ACCTGTAAATTCTGGAGTTT**C**-3′

Bold bases indicate sequence added for cloning into vectors. The 3′ guide was inserted into pSpCas9(BB)-2A-GFP (PX458) and the 5′ guide was inserted into pU6-(BbsI)-CBh-Cas9-T2A-mCherry, both obtained from Addgene. Plasmids (1 µg each) were digested with BbsI (New England Biolabs) for 30 min at 37 °C. The digested plasmids were purified using ChargeSwitch PCR Clean-Up Kit (Invitrogen). Each pair of target sequence oligos (10 µM final) was phosphorylated with T4 Polynucleotide Kinase (New England Biolabs) at 37 °C for 30 min, denatured at 95 °C for 5 min, and then slowly annealed down to 25 °C at 5 °C per minute in a thermocycler. Phosphorylated and annealed target sequence oligos were diluted 1:200 and ligated into *Bbs*I digested plasmids with T4 DNA Ligase (New England Biolabs). Plasmids were transformed into One Shot^™^ Top10 Chemically Competent *E. coli* (Invitrogen) and purified using the ZymoPURE^™^ II Plasmid Midiprep kit (Zymo Research). 1 × 10^6^ YTS cells were transfected with GFP and mCherry plasmids containing guide oligos using an Amaxa Nucleofector II transfection system. Cells were pelleted at 200 × g and resuspended in 100 µl of Amaxa transfection buffer with 5 µg of both plasmids. Cells were electroporated using program X-005 and subsequently were cultured for 48 h prior to single-cell sorting of GFP and mCherry double-positive clones into 96 well plates.

### Single-cell sorting for GFP/mCherry^+^ cells

*KIR2DL1* intermediate promoter edit clones were sorted using either a Symphony S6 or FACS Aria-II cell sorter (BD Biosciences) to deposit single GFP/mCherry double-positive cells into individual wells of 96 well plates. YTS cells that had been transfected were counted, washed twice, resuspended in sorting buffer (1 × PBS pH7.4, 1% BSA, 0.5 mM EDTA, and 25 mM HEPES), and passed through 30-μm mesh (CellTrics, Sysmex) prior to sorting to remove doublets and aggregated cells. Stringent signal pulse width gates were applied by the sorting software for further assurance of clonality. Sorted single cells were collected in 96 well plates filled with 100 µl/well of RPMI 1640 supplemented with 10% FBS and glutamine/pen/strep. Plates were monitored for growth, and clones were subsequently expanded.

### Screening of YTS intermediate promoter edit clones

Genomic DNA was isolated from the YTS intermediate edit clones with the Quick-DNA^™^ Mini Prep kit (Zymo Research). Thirty nanograms of DNA from each clone was screened by PCR using 2DL1 distal-For (5′-GGAGAGAGACAGACGGAAAAC-3′) and 2DL1 intermediate-Rev (5′-CGCTAGAATTTGACACCTAGTG-3′) primers to identify clones with homozygous or heterozygous deletions and unedited (wild-type) clones.

### Cell transfection and luciferase reporter assay

5 × 10^5^ YTS cells were seeded per well in a 24-well plate the day before transfection; 4.5 µg of reporter construct DNA, 400 ng of *Renilla* DNA, and 6 µl of jetOPTIMUS transfection reagent (Genesee Scientific) were diluted in 150 µl buffer and incubated at room temperature for 10 min before addition to each well. The DNA mixture containing plasmid DNA was then added to each well and incubated at 37 °C in 5% CO_2_ for 48 h before luciferase activity analysis. Luciferase activity was assayed using the Dual-Luciferase Reporter Assay System (Promega) according to the manufacturer’s instructions. Briefly, the culture medium was removed 48 h post-transfection, and cells were washed with 0.5 ml of phosphate-buffered saline (PBS, pH 7.4). Cells were then lysed with passive lysis buffer. The suspensions were centrifuged at 14,000 rpm for 1 min. A total of 20 μl of cell lysate supernatant was mixed with 100 μl of luciferase substrate, and the light units were measured on a luminometer. Measurements of firefly luciferase activity from promoter constructs were normalized relative to the activity of the *Renilla* luciferase produced by the pRLSV40 control vector, and each construct was tested in duplicate in at least three independent experiments.

### RNA isolation

Total RNA was isolated from YTS cells using the RNeasy^®^ Mini Kit (Qiagen). Cells were lysed in buffer RLT per manufacturer’s recommendations, and lysates were loaded on RNeasy Mini Spin Columns and washed with buffer RW1 and spun by centrifugation at 10,000 × g for 1 min. On-column DNase I digestion was performed using the RNase-Free DNase Set (Qiagen). Digested samples were washed with buffer RW1 followed by two washes with buffer RPE spinning by centrifugation at 10,000 × g after each wash. RNA samples were eluted in 25 µl of elution buffer, and RNA concentrations were determined by using a NanoDrop 2000 Spectrophotometer (Fisher Scientific).

Poly(A)^+^ mRNA was isolated using the NEBNext^®^ High Input Poly(A) mRNA Isolation Module (New England Biolabs). Fifty micrograms of purified total RNA was the input for each poly(A)^+^ mRNA sample in a 200 µl reaction. Poly(A)^+^ mRNA was captured and washed on oligo(T)_25_ paramagnetic beads provided by the manufacturer, eluted with 17 µl of Tris buffer, and quantified using a NanoDrop 2000 Spectrophotometer (Fisher Scientific). Typical yields ranged from 400 to 600 ng of poly(A)^+^ mRNA per sample.

### cDNA synthesis

cDNA samples were synthesized using TaqMan^®^ Reverse Transcription Reagents (Applied Biosystems). Each cDNA synthesis reaction was performed in a 20 µl volume with either 1 µg of total RNA or 100 ng of poly(A)^+^ RNA, 1.75 mM MgCl_2_, 2 mM dNTPs mix, 2.5 µM random hexamers, 1 U RNase Inhibitor, and 1 U of MultiScribe™ RT. The cDNA was synthesized using a 2720 Thermal Cycler (Applied Biosystems) with a single cycle of 25 °C for 10 min, 37 °C for 30 min, and 95 °C for 5 min, followed by a 4 °C hold.

### PCR of KIR2DL1 transcripts

PCR amplifications of *KIR* transcripts were performed in 40 µl reactions with ZymoTaq premix. Each PCR reaction utilized 2 µl of cDNA template, 1 µM of forward and reverse primers, and 20 µl of 2X ZymoTaq premix. Initial denaturation was performed at 95 °C for 10 min per the manufacturer’s recommendation, 30–35 cycles of 95 °C for 30 s, 30 s annealing at a primer-specific temperature, and extension at 72 °C for 0.5–1.5 min for transcripts up to 1 kb in length. A final extension was performed at 72 °C for 7 min.

### PCR primers

2DL1 exon 1-For5′-CGGCAGCACCATGTCGCTCT-3′

2DL1 exon 3-Rev5′-AGGTCCCTGCCAGGTCTTGCG-3′

2DL1 intermediate-For5′GAGTTGGTCATAGTGAAGGACACTAG-3′

2DL1 distal-For5′-GGAGAGAGACAGACGGAAAAC-3′

B-actin-For5′-CCTGGCACCCAGCACAATG-3′

B-actin-Rev5′-GGGCCGGACTCGTCATACT-3′

### KIR2DL1 detection by flow cytometry

1 × 10^6^ YTS cells were washed twice in staining buffer (1 × PBS pH 7.4 without Ca/Mg, 1% BSA, 0.5 mM EDTA, and 0.1% NaN_3_ sodium azide) by centrifugation at 100 × g for 10 min in 5-ml polystyrene round-bottom tubes (Falcon 352,052). YTS cell pellets were incubated with 1 µg PE-conjugated CD158/KIR2DL1 antibody (clone REA284, Miltenyi Biotec) for 30 min on ice, washed twice with staining buffer, gently resuspended in Cytofix™ buffer (4.2% formaldehyde, BD Biosciences), and then stored at 4 °C until analysis. Data was collected using a FACS Symphony A5 cytometer (BD Biosciences) and analyzed with FlowJo v10.8.1 (BD). Antibody binding data were expressed as % positive compared to unstained control cells; PE median fluorescence was also assessed to indicate receptor density.

### Bulk cell sorting for KIR2DL1^+^ cells

KIR2DL1^+^ cells were sorted to uniform high purity using either a Symphony S6 or FACS Aria-II cell sorter (BD Biosciences). YTS cells were counted, washed twice, resuspended in sorting buffer (1 × PBS pH7.4, 1% BSA, 0.5 mM EDTA, and 25 mM HEPES), and incubated with 10 µl PE-conjugated CD158/KIR2DL1 antibody (clone REA284, Miltenyi Biotec) for 30 min on ice. Labeled cells were washed twice with sorting buffer, resuspended in 0.5–15 ml sorting buffer, passed through 30-μm mesh (CellTrics, Sysmex), and maintained at 4 °C during sorting. Sorted KIR2DL1^+^ cells were collected in RPMI 1640 supplemented with 10% FBS and glutamine/pen/strep. Sorted cells were monitored for growth and subsequently expanded.

### Statistical analysis

Mann–Whitney *U* and two-tailed *t*-tests were performed using Prism version 9 for Mac OS; *p* < 0.05 was regarded to be statistically significant.

## Results

### Deletion of the KIR2DL1 intermediate promoter region

**Fig. 2:**
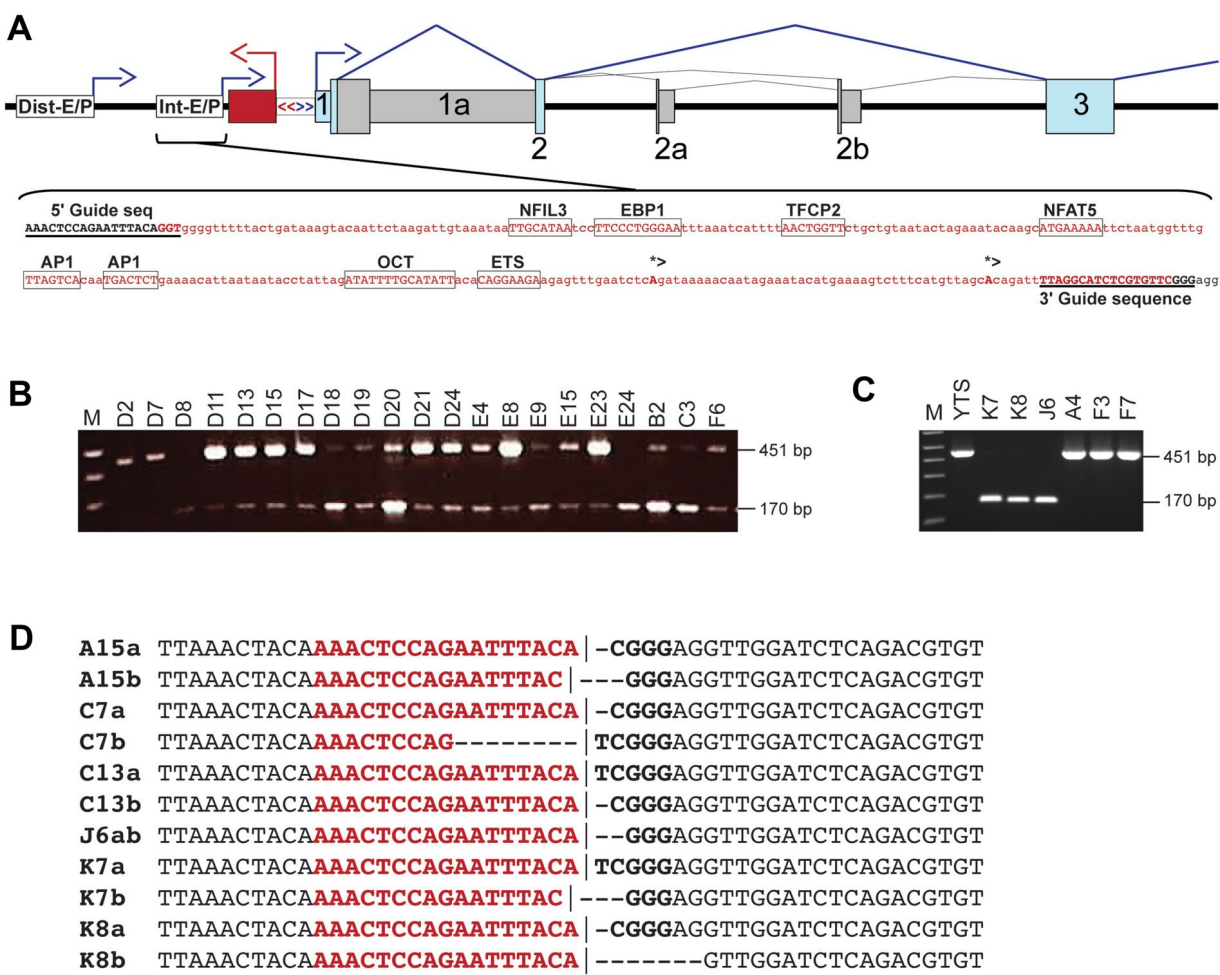
Deletion of the intermediate element from the 5′ region of the KIR2DL1 gene. **A** A schematic of the *KIR2DL1* 5′ region depicting regulatory elements and alternative exons. Sense strand exons are indicated by blue rectangles, and alternative exons are shown in gray. Open reading frames are indicated by the tall boxes. The unspliced proximal antisense transcript is shown as a red rectangle. White labeled rectangles indicate the positions of the distal (Dist-E/P) and intermediate (Int-E/P) enhancer/promoter regions. The bidirectional proximal promoter is indicated by the white rectangle with divergent red and blue arrows. The sequence of the intermediate promoter/enhancer element is shown underneath with the red type indicating the region deleted by the 5′ and 3′ CRISPR guides which are underlined in bold caps. Predicted transcription factor binding sites are in caps and boxed. Transcription start sites are in bold caps and indicated by asterisks and forward arrows. **B** PCR screening of YTS single-cell clones for the 281 bp deletion of the intermediate promoter/enhancer region. The bands corresponding to the wild-type 451 bp amplicon and the deleted ~ 170 bp fragment are indicated. **C** Re-screening of selected clones with stable expression of KIR2DL1. **D** Deletion junctions. The sequence of PCR amplicons from six homozygous deletions is shown. Individual alleles are indicated by either “a” or “b” after the clone’s name. The J6 clone had only a single junction sequence and is indicated “ab” to indicate homozygosity for this deletion. The 5′ guide sequence is shown in red bold type, and the residual 3′ guide sequence is shown in black bold type. Vertical lines indicate deletion junctions

Our previous study of the intermediate promoter regions of the *KIR* genes revealed distinct sequence elements associated with the major classes of KIR (Li et al. 2016), suggesting that this region may play a role in the tissue specificity or timing of KIR expression. To address the role of this element in KIR expression, CRISPR guides were designed to delete a 281 bp region encompassing the *KIR2DL1* intermediate promoter, including the observed transcription start sites. Figure 2A shows a schematic of the *KIR2DL1* 5′ region, including the observed retention of intron 1 and the alternative exons in intron 2 that contain stop codons. The region of the *KIR2DL1* gene targeted for deletion contains previously characterized AP1-, OCT-, and ETS-binding sites (Li et al. 2016), as well as predicted binding sites for the lineage-determining transcription factors NFIL3 and EBP1. Transfection of vectors containing both the Cas9 gene and guide RNAs into the YTS cell line resulted in the efficient deletion of the intermediate promoter element (Fig. 2B). Individual clones were isolated, and the presence of the 281 bp deletion in selected clones was verified by PCR of genomic DNA (Fig. 2C). Sequencing confirmed that the expected deletion was the only alteration in the 2 kb intergenic region. Figure 2D shows the pattern of deletions observed in 6 individual clones. The 5′ cut occurred primarily 3 bp upstream from the end of the guide sequence, and the 3′ cut varied between − 5 to + 2 bp from the end of the guide sequence.

### The KIR2DL1 intermediate element does not enhance proximal promoter activity

**Fig. 3:**
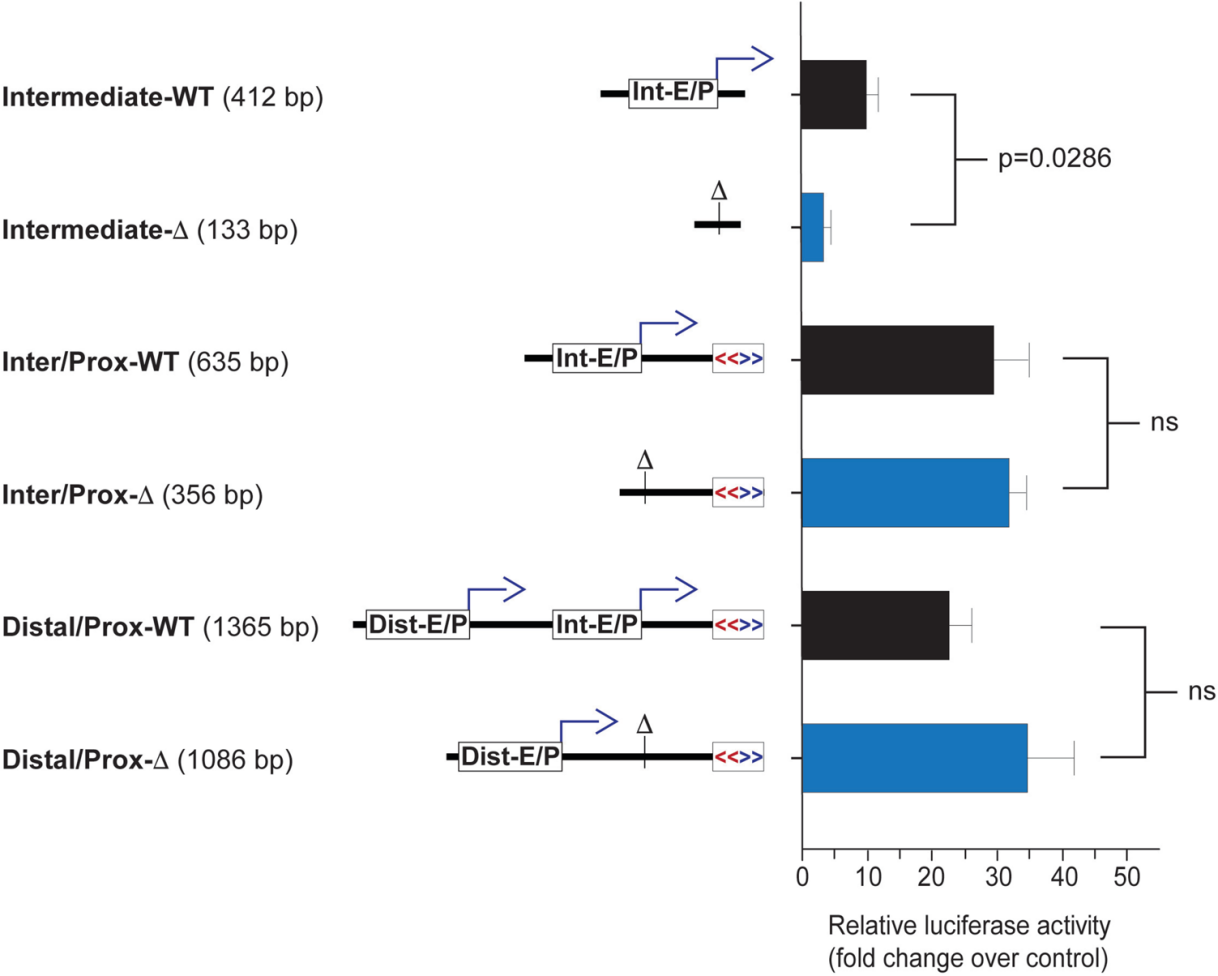
The intermediate element does not display enhancer activity. A list of pGL3 constructs containing the intermediate element and the corresponding CRISPR deletion construct is shown on the left with insert sizes in parentheses. The central region shows schematic diagrams of each construct with the distal enhancer/promoter (Dist-E/P) and intermediate enhancer/promoter (Int-E/P) elements indicated by the white rectangles with forward bent arrows. The bidirectional proximal promoter is indicated by the box containing divergent red and blue arrows. Sites of intermediate element deletion are marked by vertical lines with a ∆ symbol. The right panel shows the results of luciferase assays of transfected YTS cells. Fold change in light units relative to empty pGL3 vector is shown on the *y*-axis. Values represent the mean, and error bars indicate the SEM of at least three independent experiments. A significant *p*-value is shown for the Int-E/P constructs; ns indicates an insignificant *p*-value

Luciferase reporter assays were performed in transfected YTS cells to confirm that the intermediate promoter deletion resulted in the loss of promoter activity and to test the effect of the intermediate element on proximal promoter activity. Figure 3 shows the luciferase activity of the intermediate promoter, the combined intermediate and proximal promoters, and the full distal-intermediate-proximal 5′ region. As expected, the 281 bp deletion significantly impacted intermediate promoter activity. However, there was no loss of proximal promoter activity associated with the deletion of the intermediate element, indicating a lack of enhancer activity in the YTS cell line. In the full 5′ region construct, deletion of the intermediate region increased promoter activity, suggesting an inhibitory effect of transcription from this region on either the upstream distal promoter or the downstream proximal promoter. The increased promoter activity of the full-length construct with deletion of the intermediate element did not reach statistical significance due to a large variation in light unit activity between experiments. However, the trend of increased activity in the deleted construct as compared to the wild-type construct was maintained.

### Intermediate promoter deletion increases upstream transcripts without affecting alternative splicing

**Fig. 4:**
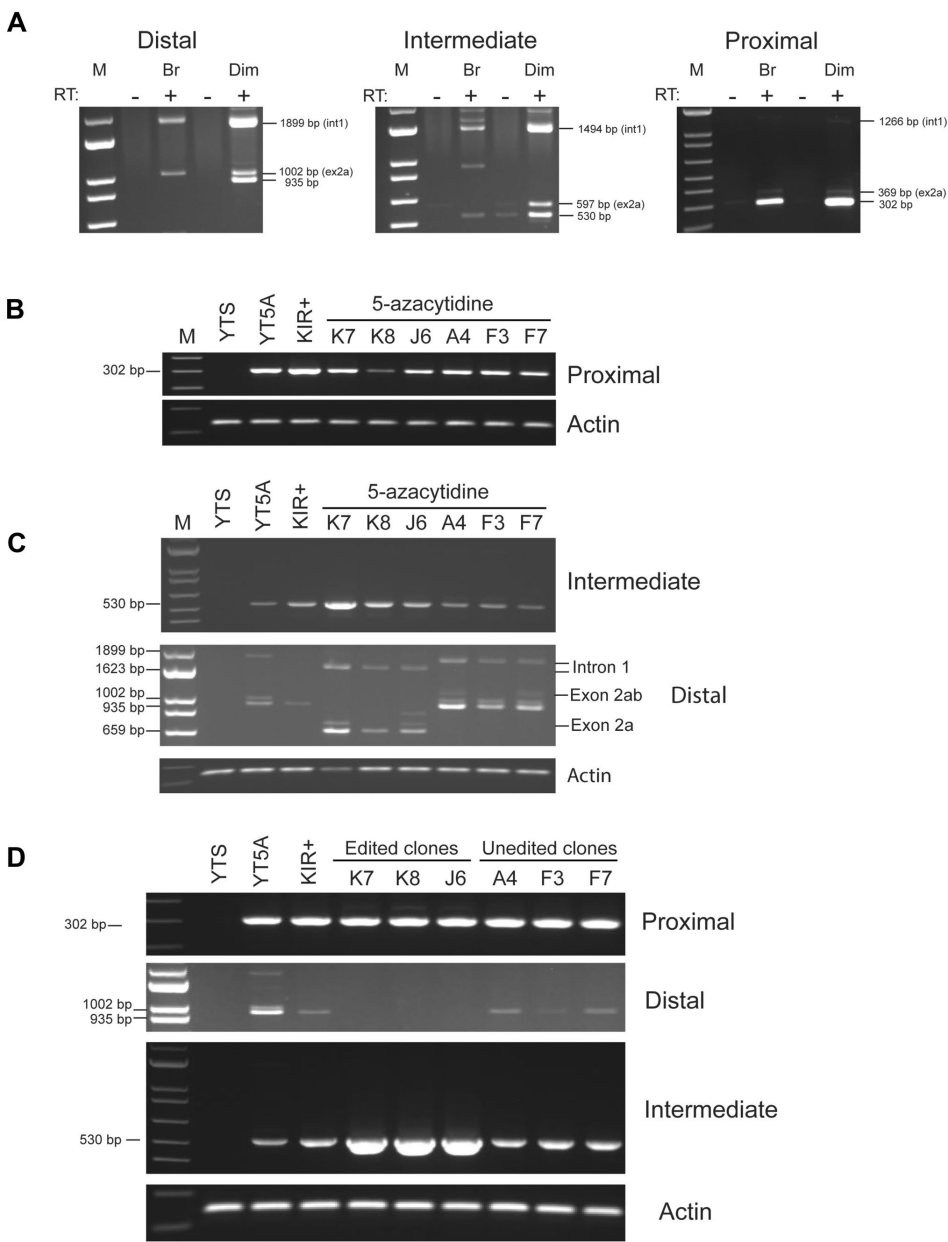
Intermediate promoter deletion does not affect distal transcription and splicing. **A** *KIR2DL1* splice forms in peripheral blood CD56^bright^ and CD56^dim^ NK cells. RT-PCR of sorted CD56^bright^ (Br) or CD56^dim^ (Dim) peripheral blood NK cells with primers specific for the distal (left panel), intermediate (center panel), and proximal (right panel) *KIR2DL1* transcripts is shown. The position of bands containing either the alternative exon 2a (ex2a) or retaining intron 1 (int1) is indicated. Control PCR reactions lacking reverse transcriptase (RT: -) are shown. All PCR products were cloned and sequenced, and the exact size in bp is indicated for each band. Data are representative of experiments performed on six individuals, all bearing only the KIR2DL1*003 allele which corresponds to the allele present in the YTS cell line. **B** 5-Azacytidine induction of proximal *KIR2DL1* transcription. RT-PCR of total RNA from parental YTS, 5-azacytidine-induced YTS (YT5A), a stable KIR2DL1-expressing cell line (KIR +), and individual CRISPR clones treated with 5-azacytidine is shown. **C** 5-Azacytidine induction of distal and intermediate *KIR2DL1* transcription. RT-PCR of polyA-selected mRNA from parental YTS, 5-azacytidine-induced YTS (YT5A), a stable KIR2DL1-expressing cell line (KIR +), and individual CRISPR clones is shown. The size of alternatively spliced mRNA containing exon 2a, exon 2a and exon 2b (exon 2ab), or retention of intron 1 (Intron 1) is shown. **D** Proximal, intermediate, and distal transcripts in stable KIR2DL1-expressing clones. RT-PCR of total RNA from parental YTS, a stable KIR2DL1-expressing cell line (KIR +), and individual CRISPR clones is shown

We had previously noted effects of the introduction of an inhibitory ZEB1-binding site on the utilization of alternative exons 2a and 2b which contain premature stop codons that would block KIR expression when included in spliced mRNAs. Decreased distal transcription in *KIR2DL1* alleles containing the ZEB1 site was associated with increased proximal transcription and inclusion of alternative exons in peripheral blood NK cells. Figure 4A shows a comparison of *KIR2DL1* transcript isoforms in immature CD56^bright^ versus mature CD56^dim^ peripheral blood NK cells from individuals bearing only KIR2DL1*003 alleles. KIR2DL1*003 is expressed by a higher percentage of NK cells than other common *KIR2DL1* alleles, and it is the allele present in the YTS cell line (Dunphy et al. 2015). CD56^bright^ NK cells had distal transcripts containing alternative exon 2b, with no detectable productively spliced *KIR2DL1* mRNA. Distal transcripts in CD56^dim^ NK cells contained both splice forms, suggesting a switch from untranslatable to productively spliced *KIR2DL1* mRNA in mature NK cells. Intermediate transcripts showed primarily productively spliced mRNAs, indicating that exon 2a inclusion is associated with transcription from the distal promoter. Figure 4B, C shows transcripts detected in YTS clones induced to express KIR2DL1 with 5-azacytidine. Consistent with the results observed in Fig. 4A, alternatively spliced *KIR2DL1* mRNAs were readily detected in distal transcripts but not intermediate transcripts. Furthermore, the deletion of the intermediate region did not affect alternative splicing, indicating that intermediate transcripts are not alternatively spliced (Fig. 4C). Although alternatively spliced mRNAs containing exons 2a and/or 2b could be readily detected in peripheral blood NK cell RNA or 5-azacytidine-induced YTS cells (Fig. 4A–C), they were not detected in YTS clones stably expressing KIR2DL1 protein, likely due to the low to undetectable levels of distal transcription (Fig. 4D). Interestingly, transcription from the region adjacent to the intermediate element was increased in stable KIR2DL1-expressing YTS clones containing the 281 bp deletion, suggesting that elements in the region flanking the deleted element contain promoter activity that was unmasked by the deletion. The increased promoter activity of the full distal–proximal construct lacking the intermediate promoter region is consistent with this result (Fig. 3). However, the enhanced transcriptional activity is not due to increased distal promoter activity since PCR with a distal primer did not detect significant levels of distal transcripts in clones with the deletion (Fig. 4D).

### Intermediate promoter deletion affects the efficiency of gene activation

**Fig. 5:**
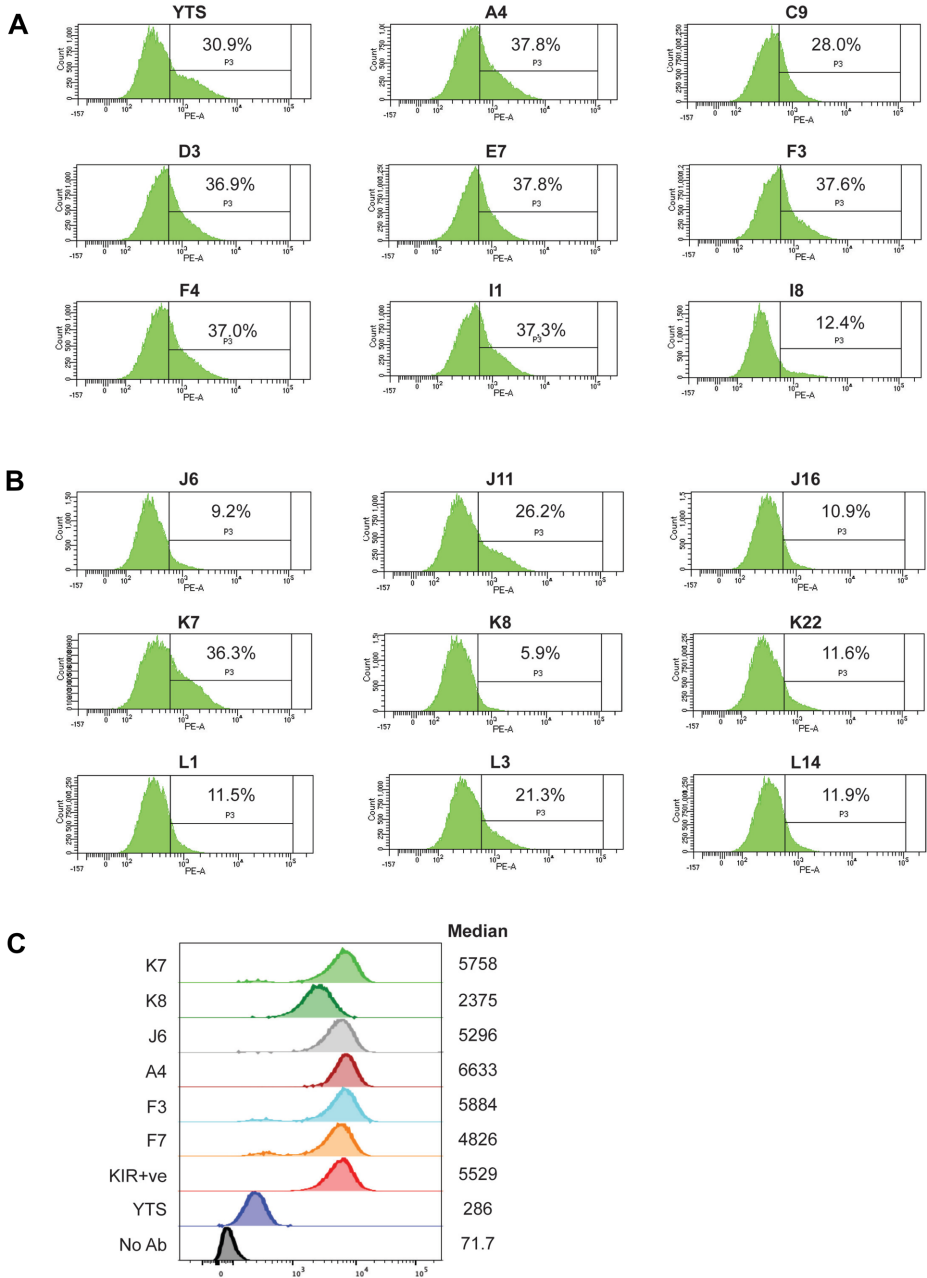
Deletion of the intermediate element reduces gene activation by 5-azacytidine. **A** 5-Azacytidine-induced expression of parental YTS cells and unedited CRISPR clones. KIR2DL1 staining of 5-azacytidine-treated cells relative to treated cells without antibody is indicated by the percentage of cells in the positively staining gate. Clone names are indicated above each panel. **B** 5-Azacytidine-induced KIR2DL1 expression of nine clones with CRISPR deletion of the intermediate element. The percentage of positive cells relative to unstained 5-azacytidine-treated cells is shown. Clone names are indicated above each panel. **C** Median KIR2DL1 expression levels of CRISPR clones with stable KIR2DL1 expression. Median fluorescence values are listed at the right of the histograms. Fluorescence values of the stable KIR2DL1-expressing cell line (KIR +), the parental YTS cell line, and the YTS cells without antibody (No Ab) are shown. Data are representative of 5 independent experiments

FACS analysis was performed on YTS clones to determine the effect of intermediate promoter deletion on gene activation and expression. The parental YTS line and 8 subclones from the CRISPR experiment with an intact intermediate region were compared with 9 subclones that had a homozygous deletion of the intermediate element. The 5-azacytidine-induced expression was significantly reduced in deleted clones relative to control clones, with one clone in the unedited group exhibiting low expression (clone I8; Fig. 5A) and one clone in the edited group that had the same level of induction as unedited clones (clone K7; Fig. 5B). These results suggest that the intermediate *KIR2DL1* element plays a significant role in the induction of gene expression. To determine if there was an effect of the deletion on the level of protein expression, KIR2DL1-expressing subclones were isolated from 5-azacytidine-induced clones. Three unedited and three edited subclones with stable expression of KIR2DL1 were isolated, and the relative levels of KIR2DL1 expression were determined. As shown in Fig. 5C, only K8 had a significant reduction in median fluorescence intensity, and it showed the least efficient induction of KIR2DL1 expression (Fig. 5B). The unusual behavior of this clone may be related to the unique 3′ deletion junction observed in this clone (Fig. 2D). The reduced expression of K8 is also consistent with an observed decrease in proximal transcripts (Fig. 4B). These results support the predicted role of the intermediate promoter in gene activation rather than acting as an enhancer for the proximal promoter.

## Discussion

Previous studies have established a central role for the *KIR* proximal promoter in the regulation of KIR expression. Methylation of the proximal promoter has been shown to represent the primary mechanism distinguishing silent *KIR* genes from those that are actively transcribed and expressed, and treatment with the demethylating agent 5-azacytidine is sufficient to induce KIR expression in NK cell lines (Santourlidis et al. 2002; Chan et al. 2003). Notably, unlike silent mouse *Ly49* genes which contain both repressive histone signatures and gene methylation (Rouhi et al. 2006; 2007), silent *KIR* genes are found in regions with active histone signatures and thus seem to be primarily controlled at the level of DNA methylation (Santourlidis et al. 2008). Although the precise mechanism controlling the differential methylation of *KIR* genes has yet to be fully defined, antisense transcription from either proximal or intronic promoters has been implicated (Cichocki et al. 2010; Wright et al. 2013). Furthermore, distal *KIR* transcription in immature NK cells and progenitors may play a role in the initial opening of the *KIR* loci (Fig. 1C; Cichocki et al. 2009). In the current study, we addressed the role of an intermediate promoter/enhancer element in *KIR* gene activation and expression. Previous analyses revealed gene-specific differences in the properties of the intermediate element among distinct *KIR* gene subfamilies, such as the *KIR2DL2*/*KIR2DL3* genes that are expressed earlier and at a higher frequency after bone marrow transplantation (Fischer et al. 2007; Li et al. 2016). Additionally, reduced distal/intermediate transcription in the KIR2DL1*0040115 allele was associated with decreased KIR protein expression, even though proximal transcription was increased, suggesting that either distal or intermediate transcripts are required for KIR expression (Wright et al. 2014). In the current study, we observe that deletion of the intermediate element does not decrease the activity of the proximal promoter, making it unlikely that the intermediate region acts as an enhancer element (Fig. 3). Although deletion of the *KIR2DL1* intermediate element in luciferase reporter constructs reduced *in vit*ro promoter activity, homozygous deletion of the element in the YTS cell line resulted in an unexpected increase in upstream transcription. It is conceivable that negative regulatory elements are present within the intermediate region. However, the observed increase in upstream transcripts exhibited by edited YTS clones stably expressing KIR2DL1 had no effect on the level of proximal transcripts or KIR protein expression compared to wild-type cells. Deletion of the intermediate element did not alter the production of alternatively spliced transcripts retaining intron 1 or exons 2a/2b, indicating that the production of untranslatable mRNAs is associated with distal rather than intermediate transcription. However, distal transcripts seem to be required, and their level is correlated with the level of proximal transcripts during 5-azacytidine-induced gene activation. While the distal transcript is low/undetectable in stable KIR2DL1-expressing cells, the highest levels of protein expression seen in 5-azacyticine treated cells in this study were observed in both the edit clone K7 and wild-type clone A4. Both clones had higher levels of distal transcript than their respective counterparts and exhibited higher levels of surface KIR2DL1 expression. The most significant effect of intermediate element deletion was on the efficiency of gene activation by 5-azacytidine-induced gene demethylation. This is consistent with our previous observation that the intermediate element is distinct in *KIR* gene subfamilies with different expression characteristics, suggesting that the principal role of this element is to initiate gene expression.

In conclusion, the current study implicates the intermediate element in the process of *KIR* gene activation and suggests that the distal element may act as an enhancer and play a role in regulating the level of KIR expression. The study of *KIR* gene regulation reveals the importance of upstream/downstream promoter elements in the initial opening of the locus, in contrast to the traditional view of a proximal promoter regulated by enhancer/silencer elements. Additional studies examining the effect of deletion of additional upstream and downstream *KIR* enhancer/promoter elements active at various stages of NK cell development will be required to achieve a complete understanding of *KIR* gene regulation.

## Data Availability

All sequence information is available in public databases. Primary data, recombinant DNA, and cell lines generated in this study are available upon request.
